# Storage Batteries and Their Uses in Dentistry

**Published:** 1894-05

**Authors:** W. W. Vance

**Affiliations:** LINCOLN, NEBRASKA.


ARTICLE V.

### STORAGE BATTERIES AND THEIR USES IN DENTISTRY.

W. W. VANCE, D. D. S., LINCOLN, NEBRASKA.

I consider the dental motor of primary value; next in importance is the laboratory motor for driving the grinding and polishing lathe; next the electric mallet, then the root canal dryer, the mouth lamp and warm air syringe or blower. As for the dental engine operated by electricity there are a number of forms, enough at any rate to satisfy the most fastidious. I prefer the motor for general use, mounted on a bracket attached to the wall or window casing. A motor thus mounted is out of the way when not in use, and does not take up any floor space. It is, when properly adjusted, as noiseless as the dental engine, and easily brought into play when needed without unnecessarily alarming the patient.

For the laboratory motor, at least a one-eighth horse-power motor should be supplied, and may be either shunt or series wound. When operated by the incandescent electric light circuit, a series wound machine is to be preferred, but for a battery circuit from a storage battery a shunt wound machine will give better results, owing to the low potential of the current. For operating such an electric plant by storage batteries would require six to twelve cells, owing to the make, as different makers and different types of cells give varying quantities of current.

The storage battery is the best for dentists, because it is the few who are so fortunately located as to have access to a city day-circuit, and it is the storage battery alone that makes it possible to avail ourselves of this current.

Of course, the first cost of such an installation is considerably more than where direct connection can be made to a circuit, but there are several advantages that the accumulator installation has that more than counterbalances the cost, and not the least of these is its absolute safety from shock if properly protected by switches or cutouts when not charging, but when the operation of charging is going on it should be disconnected from all devices by switches, and especially such as are used about the chair if a fountain spittoon, steam, water or gas pipes or gas bracket are within reach of the chair.

Large installations are best charged from a constant potential (incandescent) circuit, but small installations, such as are used by dentists, are best charged from the constant current (arc) circuit. The reason for this seeming difference exists in the fact that as the cells or accumulators each have about two volts potential, it follows that there will only be as much counter electro-motive force as there are cells multiplied by two, that is for twelve cells connected up in series, twenty-four volts. In large installations, such as are used for auxiliary lighting or for street car propulsion, a large number of cells are required, each giving two volts to the cell, this gives a counter electromotive force of a voltage approaching that of the ordinary constant potential circuit used for incandescent lighting, consequently the full force of the charging current may safely be turned into the battery without danger of damaging the plates forming the cells, and as the counter electromotive force of the cells in series is sufficient to prevent an abnormally large current from passing, it must be remembered that the charging current always should go into the battery in the opposite direction from the discharging current given off, and till the charging current has a voltage equal to and a little above the voltage of the battery no current will go into it, but in an installation of a small number of cells where the voltage of the battery is not close to 100 volts, the incandescent current, such as the Edison and similar circuits of about 110 volts, would charge too rapidly and ruin the battery by warping the plates, as it is the quantity of current that passes through the battery that does the harm. However, while the voltage of the incandescent current is low as compared with the arc light circuit, the quantity is constant in the latter and many times less than the incandescent circuit; and while the voltage of the constant current (arc) circuit may vary according to the number of lights burning, which is usually from 1,500 to 3,000 volts, yet the quantity is practically unvarying, and is only about nine to twelve amperes. This being all there is of it, it can all pass through the battery without doing any harm, and the variation of voltage cuts no figure because the quantity is limited. These are briefly the reasons for using the arc circuit for charging small installations of accumulators.

We are apt to measure others by our own standard, and I beg your indulgence if I mention my reasons and point out difficulties which experience has proven to me exist, but that were not so manifest till after I had learned by trial that better things and methods exist than we have been using, and flattered ourselves were very good and rested content to use, till competition forces us to investigate and adopt better methods. In the first years of my practice I depended on the ordinary dental engine, and considered it a great advance over the long handled burs and drills rotated between thumb and finger with the end of the handle resting in a bur thimble supported by a ring over one of the fingers and the socket in the palm of the hand. Such methods were used by my preceptor when I first commenced the study of my profession, but were soon after discarded for the more modern method known as the dental engine. Finally I thought that even that instrument could be improved, and some of the disadvantages it possessed might be wholly or partly eradicated. Among those disadvantages were the contortions we were obliged to assume to see our cavity and at the same time tread the engine (for you must remember that all did not have the extremely flexible Johnston spring at first); then a high speed was not obtainable without considerable effort. This caused unsteadiness of the hand. The engine was almost of necessity placed at the right of the patient and a little in front of the operator, a position many times awkward and unsatisfactory.

All of these things I readily saw could be eliminated by the use of an external application of power, and especially if the motive power could be placed or brought to a position directly in front of the patient and made capable of an upward and a downward movement, and also adjustable either to the right or left, sufficiently to accommodate the position of the operator's arm. The electric motor solved this problem directly, and only had the disadvantage of needing intelligent and painstaking care of the battery. This care and attention, however, brought, by reason of occasional neglect and carelessness, unreliable results, and sometimes the motor or mallet would not work. Here, then, was a difficulty unlooked for, which soon brought the whole into disfavor, not alone from those who met disappointment, but by others who "suffer the ills they had rather than flee to others they knew not of," but had received descriptions of from their unfortunate brothers. The direct current when obtainable seemed to offer the best solution of this battery difficulty, and for laboratory use this is undoubtedly so yet. But for operations at the chair new difficulties arose. Primary batteries were always of low potential, and there was no difficulty from shock, but not so with the dynamo current of 110 volts potential and upward, for they frequently, and in practice, are almost invariably grounded, a fact which always rendered its use at the dental chair likely to give a slight shock to the patient if favorable conditions chanced to exist. One of these was the use of a fountain spittoon attached to the chair, and especially on damp days. Gas, water and steam pipes in such proximity to the chair as likely to be touched by the patient was another. All of these difficulties are avoided absolutely by the agency of the storage battery.

For operating the miniature lamps, the hot air syringe and root canal dryer, while it is possible to do so with the direct current from the dynamo furnishing current for incandescent lighting and power, it is not the best, because the heat generated in the controlling device is too great to be conveniently disposed of except by converting the incandescent current into motion by means of a motor, and this motion of power back into current of low potential and great quantity by means of a properly constructed dynamo, either mounted on the same base with the motor, or driven by it by means of a belt. Such a dynamo need not be larger than an ordinary one-eighth horse power motor, and can be obtained from any manufacturer of motors; the type can be the same as the motor, the difference being in the winding. The storage battery solves all of these difficultes, viz., no shock, for if ordinary care is taken there need be no "ground;" large quantity and low potential, as perfect control as the most exacting could possibly wish, and last, but not least, good and reliable service with a minimum amount of care.

It is not within the province of this paper to suggest any particular battery, but my own observations along this line lead me to believe that we may expect some very excellent results in storage batteries and great improvements in the next year or two. But if any are contemplating adopting electricity, my advice is not to wait; the extra expense incurred by any change in your battery will be more than made up by the good you obtain by early adoption.

A few suggestions may not be amiss in regard to your installation. Locate your battery in a convenient and sufficiently light place, so as to be easy of access and inspiration. Inspect it every day, and always before commencing to charge. See that the active material in the plates does not fall out and accumulate at the bottom of the cells, so as to touch the plates; this would cause a short circuit and injure the plates, also diminish the power of cells. Use well-insulated copper wire, of not less than No. 15 B. & S. gage, and No 12, especially if the battery is located more than twelve or fifteen feet away from your appliances at the chair. The reason for this is that the quantity of your current should not be wasted in traversing an unnecessary length of wire, or the force or voltage of the current reduced by the high resistance of small wire external to the magnet coils of your motor or mallet; this loss is reduced toward the minimum just in proportion to the increase in size of the conducting wires leading from the battery to the motor.

The Donaldson-Macræ battery comes as near fulfilling the requirements of the most exacting demands of dentists as anything I have investigated, and has some special claims not made for any other storage batteries I have had the privilege of investigating. Among these is the impossibility for the active material to fall out and accumulate at the bottom of the cells; impossibility for the plates to warp, and the fact that the amount of charge remaining in a cell at any particular time can be very nearly calculated by simply measuring the specific gravity of the fluid in the cells, They are put up in a convenient form, and have a capacity sufficiently large, so that the least number of cells indicated in the earlier part of this paper will be amply sufficient to operate a one-eighth power motor, together with such other electrical apparatus as the dentist will require.—*Dental Review*.

